# Weakened untuned gain control is associated with schizophrenia while atypical orientation-tuned suppression depends on visual acuity

**DOI:** 10.1167/jov.23.2.2

**Published:** 2023-02-01

**Authors:** Victor J. Pokorny, Michael-Paul Schallmo, Scott R. Sponheim, Cheryl A. Olman

**Affiliations:** 1 Minneapolis Veterans Affairs Health Care System, Minneapolis, MN, USA; 2 Department of Psychiatry and Behavioral Science, University of Minnesota, Minneapolis, MN, USA; 3 Department of Psychology, University of Minnesota, Minneapolis, MN, USA

**Keywords:** schizophrenia, visual acuity, psychophysics, suppression, biological relatives, bipolar disorder

## Abstract

Perceptual distortions are core features of psychosis. Weakened contrast surround suppression has been proposed as a neural mechanism underlying atypical perceptual experiences. Although previous work has measured suppression by asking participants to report the perceived contrast of a low-contrast target surrounded by a high-contrast surround, it is possible to modulate perceived contrast solely by manipulating the orientation of a matched-contrast center and surround. Removing the bottom-up segmentation cue of contrast difference and isolating orientation-dependent suppression may clarify the neural processes responsible for atypical surround suppression in psychosis. We examined surround suppression across a spectrum of psychotic psychopathology including people with schizophrenia (PSZ; *N* = 31) and people with bipolar disorder (PBD; *N* = 29), first-degree biological relatives of these patient groups (PBDrel, PSZrel; *N* = 28, *N* = 21, respectively), and healthy controls (*N* = 29). PSZ exhibited reduced surround suppression across orientations; although group differences were minimal at the condition that produced the strongest suppression. PBD and PSZrel exhibited intermediate suppression, whereas PBDrel performed most similarly to controls. Intriguingly, group differences in orientation-dependent surround suppression magnitude were moderated by visual acuity. A simulation in which visual acuity and/or focal attention interact with untuned gain control reproduces the observed pattern of results, including the lack of group differences when orientation of center and surround are the same. Our findings further elucidate perceptual mechanisms of impaired center-surround processing in psychosis and provide insights into the effects of visual acuity on orientation-dependent suppression in PSZ.

## Introduction

Perceptual distortions are a primary symptom of psychosis. In 2005, Dakin, Carlin, and Hemsley (2005) reported a striking reduction of contrast surround suppression in patients with schizophrenia (PSZ), which has been borne out in several other reports (Chen, Norton, & Ongur, 2008; Schallmo, Sponheim, & Olman, 2015; Serrano-Pedraza, Romero-Ferreiro, & Read, 2014; Seymour, Stein, Sanders, Gugenmos, Theophil, & Sterzer, 2013; Tadin, Kim, Doop, Gibson, Lappin, Blake, & Park, 2006; Tibber, Anderson, Bobin, Antonova, Seabright, Wright, Carlin, Shergill, & Dakin, 2013; Yoon, Maddock, Rokem, Silver, Minzenberg, Ragland, & Carter, 2010). The magnitude of the reduction appears to fluctuate with symptom severity (Yang, Tadin, Glasser, Hong, Blake, & Park, 2013), with more recent studies in out-patient populations estimating smaller effect sizes than reported for the in-patient sample of the 2005 study. Still, the task is valuable because it quantifies the function of well-understood neural mechanisms in primary visual cortex within patient populations. In addition, because perceptions reported during the task by PSZ more closely match the physical reality of stimuli, concerns about generalized cognitive deficits impairing performance are diminished.

Several neural mechanisms – occurring both inside (i.e. intrinsic) and outside (i.e. extrinsic) of primary visual cortex (V1) work together to determine perceived contrast, which can generally be predicted from firing rates of neurons in V1 (Boynton, Demb, Glover, & Heeger, 1999). One such mechanism is V1-intrinsic untuned gain control, which provides orientation-insensitive suppression that is stronger for more intense (i.e. higher contrast) stimuli (Carandini & Heeger, 2011; Mély, Linsley, & Serre, 2018). Untuned gain control is thought to be weaker in PSZ (Butler, Silverstein, & Dakin, 2008). In addition to untuned gain control, orientation-dependent mechanisms suppress surrounds that are parallel (or near parallel) to the center whereas surrounds orthogonal (or near orthogonal) to the center produce little to no suppression (Cavanaugh, Bair, & Movshon, 2002; Mély, Linsley, & Serre, 2018; Shushruth, Nurminen, Bijanzadech, Ichida, Vanni, & Angelucci, 2013). There is some evidence that the efficacy of these mechanisms differs for PSZ (Rokem, Yoon, Ooms, Maddock, Minzenberg, & Silver, 2011), although also see other reports (Schallmo, Sponheim, & Olman, 2015).

Feedback from higher areas in the visual cortex is also known to alter firing rates of neurons in primary visual cortex (Shushruth et al., 2013; Sillito & Jones, 2002). In particular, electrocorticography measurements in human V2 and V3 (Self, Peters, & Possel, 2016) and primate electrophysiology measurements in V1 (Lamme, Rodriguez-Rodriguez, & Spekreijse, 1999) provide evidence that V1-extrinsic segmentation cues (i.e. object boundaries) modulate V1-intrinsic suppression mechanisms. For example, suppression for parallel surrounds occurs within 50 ms of stimulus onset (Bijanzadeh, Nurminen, Merlin, Clark, & Angelucci, 2018); however, when a boundary is present, neural responses occurring more than 100 ms after stimulus onset have the same amplitude for both parallel and orthogonal surrounds (Self, Peters, & Possel, 2016). This suggests a later abolishment of earlier suppression induced by the parallel surround when a boundary is present. Such an effect is thought to be mediated via feedback, possibly from border-ownership processes in V2 (Zhaoping, 2005) or V4 (Franken & Reynolds, 2021) and may be subject to regulation by attention or awareness. V1-extrinsic mechanisms are likely important to consider in the context of psychosis due to well-documented attentional deficits and evidence of altered top-down regulation of low-level inputs (Gold, Fuller, Robinson, Braun, & Luck, 2007; Gold, Robinson, & Leonard, 2018; Mathalon, Heinks, & Ford, 2004; Pokorny & Sponheim, 2021; Pokorny, Espensen-Sturges, Burton, Sponheim, & Olman, 2020; Poltoratski, Ling, McCormack, & Tong, 2017).

The present study implemented a novel contrast-matched surround suppression paradigm that manipulated relative center-surround orientation (0 degrees, 20 degrees, 45 degrees, 70 degrees, or 90 degrees) and distance (near versus far surround conditions with inner radius at 1 degree and 2.5 degrees, respectively, around a central target with a radius of 0.75 degrees). The goal was to collect the behavioral data necessary to determine whether perceptual contrast surround suppression deficits associated with schizophrenia might be attributed solely to orientation-insensitive, V1-intrinsic suppression mechanisms (untuned gain control) or whether orientation-dependent mechanisms are also altered. In the event that we observed alterations of orientation-dependent suppression, we included a far-surround condition to enable further discrimination between V1-intrinsic and V1-extrinsic mechanisms. The surround receptive field can be disaggregated into two regions (termed near and far surround) that are mediated by different neural mechanisms. The far surround is mediated by feedback connections extrinsic to V1, whereas the near-surround is mediated by a combination of both feedback connections and V1-intrinsic horizontal connections (Bair, Cavanaugh, & Movshon, 2003; Shushruth et al., 2013). Thus, by assessing contrast surround suppression for both near and far surrounds, we aimed to further distinguish the possible neural mechanisms by which PSZ experience weakened surround suppression.

Typically, contrast surround suppression is measured with a low-contrast target embedded in a high-contrast surround, but, in the present study, the luminance contrasts of the center and surround gratings were matched. Although this choice reduces the expected magnitude of the behavioral effect (Xing & Heeger, 2001), it also controls for the bottom-up contrast-difference cues that help draw spatial attention and thus is useful for clarifying whether deficits in PSZ are driven by altered attentional or low-level visual processes (Zenger-Landolt & Koch, 2001).

It is unclear whether atypical surround suppression is present in other psychotic disorders, such as bipolar disorder (Dakin, Carlin, & Hemsley, 2005; Schallmo, Sponheim, & Olman, 2015; Yang, Tadin, Glasser, Wook Hong, Blake, & Park, 2013). Given the criticisms of reliability and validity of categorical Diagnostic and Statistical Manual of Mental Disorders (DSM) diagnoses and the shared features of schizophrenia and bipolar disorder (Kotov, Krueger, Watson, Achenbach, Althoff, Bagby, Brown, Carpenter, Caspi, Clark, Eaton, Forbes, Forbush, Goldberg, Hasin, Hyman, Ivanova, Lynam, Markon, Miller, Moffitt, Morey, Mullins-Sweatt, Ormel, Patrick, Regier, Rescorla, Ruggero, Samuel, Sellborn, Simms, Skodol, Slade, South, Tackett, Waldman, Waszczuk, Widiger, Wright, & Zimmerman, 2017; Markon, Chmielewski, & Miller, 2011), understanding the degree to which visual processing impairments are reflective of categorical differences between disorders – as opposed to being reflective of a unified spectrum of psychotic experiences – may help clarify diagnostic and etiologic ambiguity. Furthermore, it is unclear whether such impairments are specific to the patient groups or extend to unaffected first-degree relatives (Greenwood, Shutes-David, & Tsuang, 2019; Pokorny, Lano, Schallmo, Olman, & Sponheim, 2021; Schallmo, Sponheim, & Olman, 2013). Given the shared genetic predisposition among patients and their first-degree relatives, common visual processing impairments would suggest such impairments to be an underlying risk factor rather than simply a consequence of having the disorder. Thus, by including first-degree relatives, we hoped to be able to better characterize the causality of the relationship between psychotic psychopathology and visual processing deficits.

Based on previous work with a similar transdiagnostic outpatient sample (Schallmo, Sponheim, & Olman, 2015), we hypothesized that PSZ would exhibit weakened orientation insensitive untuned gain control and broadened tuning width of the orientation-dependent mechanisms relative to controls. Additionally, we hypothesized that people with bipolar disorder (PBD; relative of a patient with PSZ [PSZrel], and relative of a patient with bipolar disorder [PBDrel]) would exhibit intermediate gain control and orientation-dependent suppression deficits consistent with a spectrum of psychotic psychopathology that spans conventional diagnoses reflecting that these groups share some underlying etiology with PSZ yet experience less severe phenomenological and functional disturbances. Finally, we hypothesized that PSZ would exhibit weakened suppression for both near and far surrounds suggesting a combination of impaired V1-extrinsic and V1-intrinsic mechanisms. Thus, the goals of the study were to separately characterize (1) orientation-insensitive gain-control, (2) orientation-dependent suppression magnitude and tuning width, and (3) the differential functioning of these mechanisms for near and far surrounds across a spectrum of psychotic psychopathology.

## Methods

Patients were recruited from Minneapolis Veterans Affairs Health Care System (MVAHCS) outpatient clinics, community support programs for the mentally ill, and county mental health clinics. First degree relatives of PSZ and PBD were identified by research staff using a pedigree form completed through interviews with patients and were invited by mail and telephone to participate in the study. Healthy controls (HCs) were recruited via posted announcements at fitness centers, community libraries, the MVAHCS, and newsletters for veterans. Potential PSZ, PBD, and HC were excluded if they met any of the following criteria: English as a second language, age >60 years, IQ <70, substance dependence within the past 6 months, substance abuse within 2 weeks of testing, head injury with skull fracture or substantial loss of consciousness (i.e. loss of consciousness >30 minutes), electroconvulsive therapy, amblyopia untreated before 18, epilepsy, stroke, or other neurological conditions. Additional exclusion criteria for HC were first-degree family history of major depressive disorder or a psychotic disorder (e.g. schizophrenia and/or bipolar disorder). PSZrel and PBDrel were excluded only if they had a medical condition that prevented participation.

Participants provided written informed consent before participating in the study. The study protocol was approved and monitored by the MVAHCS and the University of Minnesota Institutional Review Board and adhered to the Declaration of Helsinki. Participants were administered the Structured Clinical Interview for the DSM-IV-TR Axis-I Disorders-Patient Edition (SCID-I/P; First, Spitzer, Gibbon, & Williams, 2002), Brief Psychiatric Rating Scale, 24-item (BPRS; Overall & Gorham, 1962), Sensory Gating Inventory (SGI; Hetrick, Erickson, & Smith, 2012), and Wechsler Adult Intelligence Scale, Third Edition (WAIS-III; Wechsler, 1997). A minimum of two trained raters (advanced doctoral students in clinical psychology, postdoctoral researchers, or licensed psychologists) reached consensus on all diagnoses, based on the DSM-IV-TR criteria (American Psychiatric Association, American Psychiatric Association Staff, American Psychiatric Association 2000). Additional participant and study information is detailed in previous publications (Longenecker, Pokorny, Kang, Olman, & Sponheim, 2021; Pokorny & Sponheim, 2021; Pokorny et al., 2020). Group demographics for participants meeting inclusion criteria are tabulated in Table 1.

**Table 1.** Demographic and symptom severity information.

Variable	PSZ *n* = (31)	PBD *n* = (29)	HC *n* = (29)	PSZrel *n* = (28)	PBDrel *n* = (21)	Statistic
Percent female	32	38	52	61	52	χ²(4) = 6.269, *p* = 0.18, Cramer's V = 0.21
Age	46.42 (8.99)	47.41 (10.22)	46.24 (9.63)	47.18 (8.78)	40.52 (11.67)	F(4, 133) = 1.9, *p* = 0.11, η² = 0.05
Visual acuity (logMAR)^*^	0.14 (0.15)	0.12 (0.12)	0.08 (0.13)	0.1 (0.12)	0.04 (0.1)	F(4, 133) = 2.36, *p* = 0.06, η² = 0.07
Years education	13.32 (1.76)	15.14 (2.29)	15.9 (1.47)	14.79 (2.23)	14.86 (1.93)	F(4, 133) = 6.9, *p* < 0.001, η² = 0.17
Parental education (ranking)^†^	4.97 (1.43)	4.59 (0.8)	5.52 (1.24)	5.07 (0.86)	5.14 (0.91)	F(4, 130) = 2.59, *p* = 0.04, η² = 0.07
Estimated IQ^‡^	100.55 (13.68)	101.9 (12.92)	116.07 (13.89)	107.36 (16.9)	109.29 (15.86)	F(4, 133) = 5.31, *p* < 0.001, η² = 0.14
CPZ equivalent^§^	13.47 (23.05)	2.44 (2.04)	–	12.16 (10.99)	–	
BPRS total^ǁ^	41.74 (12.05)	37.31 (9.73)	26.07 (2.27)	32.18 (8.76)	32.1 (7.6)	F(4, 133) = 13.35, *p* < 0.001, η² = 0.29
BPRS positive	10.26 (5.19)	5.97 (1.64)	5.03 (0.19)	5.93 (2.71)	5.71 (2.61)	F(4, 133) = 14.35, *p* < 0.001, η² = 0.3
BPRS negative	4.03 (1.74)	3.9 (1.5)	3.28 (0.7)	3.36 (1.1)	3.67 (1.46)	F(4, 133) = 1.75, *p* = 0.14, η² = 0.05
BPRS disorganized	7.16 (2.54)	6.45 (1.82)	4.45 (0.78)	5.86 (2.01)	5.29 (1.52)	F(4, 133) = 9.18, *p* < 0.001, η² = 0.22
SGI total^¶^	74.63 (36.46)	79.96 (33.79)	26.96 (18.2)	50.3 (31.72)	43.58 (32.61)	F(4, 126) = 13.87, *p* < 0.001, η² = 0.31

* LogMAR: Normal is 0.0, better than normal is <0.

† Parental Education ranking: 1 is seventh grade or less, 7 is graduate or professional degree, the greater of the mother's or father's ranking is reported.

‡ IQ was estimated using the Block Design and Vocabulary subtests of the Wechsler Adult Intelligence Scale, Third Edition.

§ CPZ equivalent was computed only for the subset of participants on antipsychotics: 26 PSZ, 17 PBD, and 3 PSzrel.

ǁ Brief Psychiatric Rating Scale (BPRS) score ranges: Total, 24 to 168; Positive, 5 to 35; Negative, 3 to 21; and Disorganized, 4 to 28.

¶ SGI total range of scores: 0 to 180.

For each measure, potential group differences were assessed by 1-way ANOVA. Any measure that showed a potential group difference (using a conservative threshold of *p* < 0.1) was then tested for a relationship to the visual behavioral task using Pearson's correlation against the parallel surround condition. The only measures that showed a correlation with surround suppression were visual acuity and estimated IQ (see Supplementary Figures S3, S5). For patients, medication (converted to chlorpromazine [CPZ] equivalent; Andreasen, Pressler, Nopoulos, Miller, & Ho, 2010) was also tested for association with performance on the surround suppression task, and no association was found (r(41) = 0.16, *p* = 0.298).

**Figure 1.**
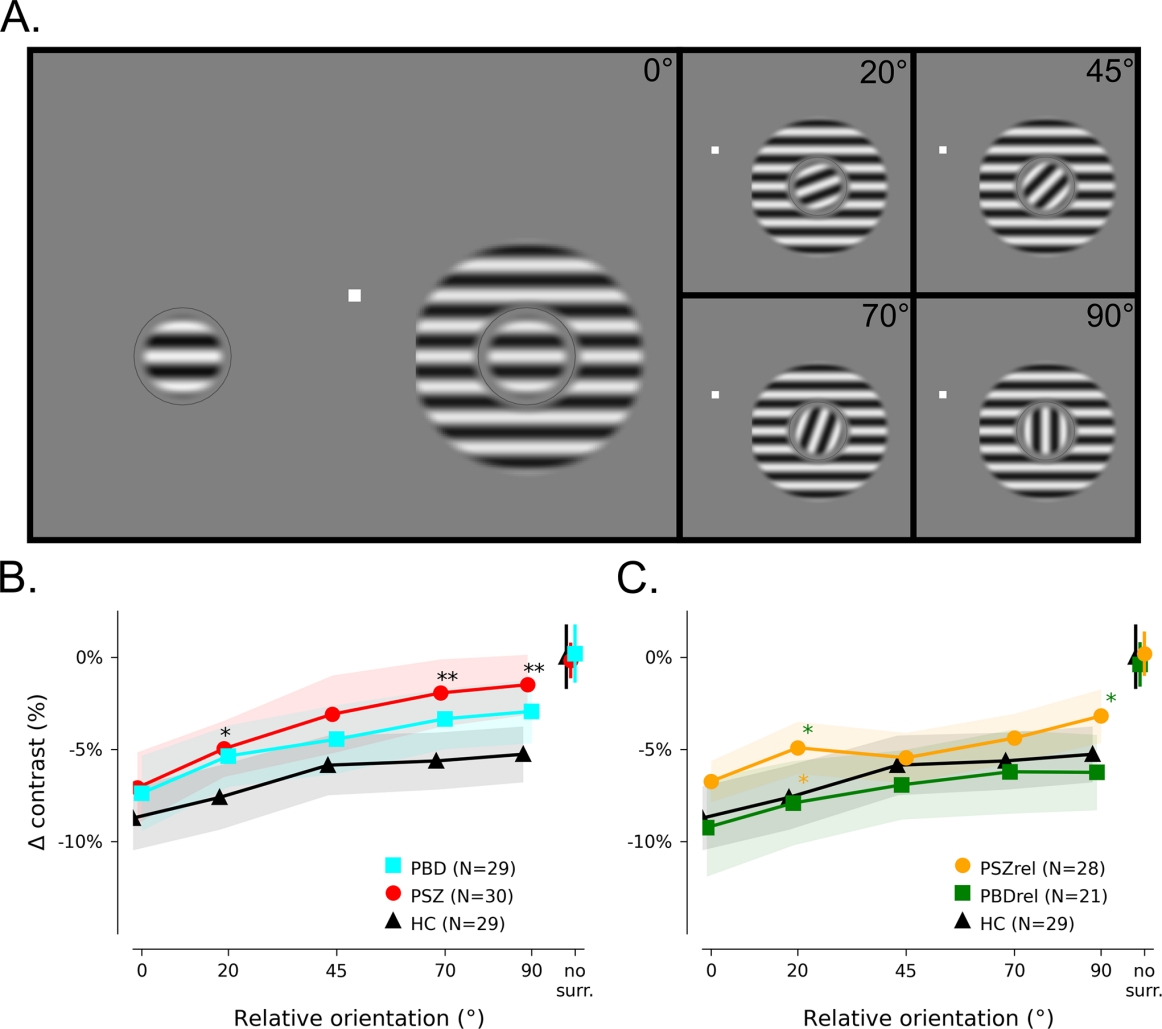
**Stimulus presentation paradigm and behavioral results.** (**A**) Near surround condition stimuli: the target (0.75 degrees radius grating with spatial frequency of 2 cycles/degrees) is separated by 0.25 degrees from a surrounding annulus with an outer radius of 2.0 degrees. All gratings appeared simultaneously and were present for 250 msec; relative orientation of target and surround was set to one of five values, but the orientation of the target grating was randomly selected on each trial. Participants had unlimited time to respond with a button press to indicate whether the circular grating on the left or right appeared to have higher contrast. (**B,** **C**) Each point represents the average contrast decrement applied to the reference grating to match the perceived contrast of the target grating. Panels **B** and **C** are the same except panel **B** depicts the patient and control groups whereas panel **C** depicts the first-degree relative and control groups. Points with error bars indicate contrast settings for a no-surround control condition. Error bars and shaded regions represent bootstrapped 95% confidence intervals. Asterisks indicate significant differences in post hoc t-tests (*: *p* < 0.05; **: *p* < 0.01); color of *asterisk* indicates group against which significant difference was measured. The same control group is presented in both plots as a reference.

All participants completed a contrast-matching task (Figure 1; task details in the legend) to assess the perceived contrast of a 1.5 degree-diameter circular grating patch presented at 3 degrees eccentricity. Before task administration, visual acuity was measured in the same room at 2 meters viewing distance (LIGHTHOUSE Distance Visual Acuity Test, Long Island City, NY). For the task, gratings were presented in three configurations: with no surround, with an adjacent surround (at 5 different relative orientations of surrounding gradings ranging from 0 degrees to 90 degrees), and with a far surround (also at 5 relative orientations). Visual stimuli were displayed on an NEC 17 inchCRT monitor (35.1 × 26.7 cm, 1024 × 768 pixels) viewed from 61 cm. The display was calibrated to produce a linear relationship between pixel intensity value (0–255) and luminance (mean luminance 102 cd/m^2^). Stimuli were generated using PsychoPy (Peirce, 2007). Target stimuli were sinusoidally luminance-modulated gratings with a spatial frequency of 2 cycles/degree, masked by a circular aperture with 0.75 degrees radius, with edges defined by a raised cosine function. Luminance contrast of the target gratings was 80%. Targets were located at 3 degrees eccentricity, 16 degrees of polar angle below the horizontal meridian (so stimuli would have an asymmetric cortical representation to permit future electroencephalogram [EEG] data analysis, not presented here). Targets were surrounded by a black circle, 1 pixel wide, that was present throughout the experiment, to remove uncertainty about target location and to aid visual segmentation of targets from surrounds. In the near surround condition, the surround stimulus was also a sine-wave grating, 2 cpd and 80% contrast, masked by an annulus with inner radius of 1 degree (i.e. 0.25 degrees gap between target and surround) and an outer radius of 2 degrees. For the far surround condition, inner and outer radii were 2.5 degrees and 5.0 degrees, respectively. Because targets were centered at 3 degrees eccentricity, surround gratings were masked (hard edge) so they did not come within 0.5 degrees of the vertical meridian or cross into the other visual hemifield. A white fixation square subtending 0.2 degrees of visual angle was present throughout the experiment.

A single trial consisted of the simultaneous presentation of 3 elements for 250 msec: a reference circular grating with no surround, a target circular grating, and an annulus surrounding the target grating (either near or far, at 1 of 5 possible relative orientations). The target and reference gratings were presented at a randomly selected orientation (0 degrees, 45 degrees, 90 degrees, or 135 degrees) on each trial; the orientation of the target and reference was the same on a given trial. The orientation of the surround was controlled relative to the target surround. There was also a “no surround” condition in which both sides of the screen appeared identical (i.e. only the target and the reference elements appeared).

The contrast of the target stimulus was always 80%; the contrast of the reference stimulus was adjusted to achieve a match in perceived contrast. The side on which the reference stimulus was presented was randomized, so the target (plus surround) occurred on both sides of the screen with equal probability. Participants responded with a two-button button box to indicate whether the circular patch on the left side or the right side of the screen appeared to have higher contrast.

For each condition, the contrast of the reference grating was controlled by a separate Psi staircase (Wichmann & Hill, 2001) implemented in PsychoPy (Peirce, 2007; Peirce, 2008; version 1.85.2) with the following parameters: alpha (threshold) range/precision [−40, 20]/1; beta (slope) range/precision [0.1, 5]/0.05; intensity (delta-contrast for reference) range/precision [−75, 15]/1; step type: linear; delta: 0.08 (lapse rate: 4%). Each staircase converged at a point of subjective equality between the reference grating (for which contrast was varied) and target (fixed contrast) grating.

Catch trials were also embedded in the task (8% of trials were catch trials). There were 48 catch trials, evenly divided between parallel and orthogonal surrounds and randomly assigned to the near or far condition. On a catch trial, the reference contrast was fixed at 30%. On these trials, participants should have always pressed the button that indicated that the non-reference side (target with surround) was of higher contrast. Performance on catch trials was used to assess participant engagement in the task and compliance with task instructions.

One experimental run contained 48 staircase trials for each condition. Conditions were not blocked; trials from different conditions were mixed together because the task never changed. Participants completed one experimental run each, providing a single estimate of perceived contrast for each of the 11 conditions (target with no surround, 5 near surround conditions, and 5 far surround conditions). The experimental run was paused four times so participants could rest their eyes and adjust their seat, verbally telling the experimenter when they were ready to continue.

## Analysis

Data from each participant were analyzed if they met the following criteria: accuracy on catch trials was better than 75% and their behavior indicated that perceived contrast of the target with near surround at 0 degrees and 20 degrees relative orientation was reduced. This last criterion was in place to eliminate participants who could not selectively attend to the central targets and instead reported the overall (target plus surround) pattern. A total of 30 datasets were discarded because they did not meet these criteria (3 PSZ, 3 BPD, 1 HC, 1 PSZrel, and 0 PBDrel because of performance on catch trials and 9 PSZ, 2 PBD, 6 HC, 4 PSZrel, and 1 PBDrel for reporting high contrast in parallel conditions), leaving a total of 138 datasets for analysis (31 PSZ, 29 PBD, 29 HC, 28 PSZrel, and 21 PBDrel).

For each of the 11 conditions (no-surround, and 5 relative orientations for each of the near and far surround conditions), perceived contrast was calculated as the mean of the last 3 threshold estimates produced by the Psignifit staircase (Schütt, Harmeling, Macke, & Wichmann, 2016) for that condition (excluding catch trials). The adaptive staircase failed to converge for one individual from the PSZ group for the no-surround trials which resulted in an outlier threshold value greater than five standard deviations from the mean for that condition only. We excluded that individual from analyses in which the no-surround threshold values were dependent variables, but included their data for all other analyses.

Following the a priori hypothesis that near and far surround suppression are mediated by different neural mechanisms, separate repeated measures ANCOVAs (rmANCOVAs) were performed to assess main effects of group and three of the surround conditions (0 degrees, 90 degrees, and no-surround) and interaction between group and surround condition while controlling for visual acuity. Although we ultimately decided to include visual acuity as a covariate, such a decision is determined by theoretical perspective (e.g. “are visual impairments an integral component of schizophrenia or simply a confounding factor?”). Given the lack of certainty around this issue, we also report our main results without including acuity as a covariate (Table 2). Additionally, the choice of including fewer conditions for the rmANCOVA was driven by the fact that as the number of levels of the within-subjects factor (i.e. number of conditions) increases, the power for detecting an effect decreases. Thus, choosing fewer levels that maximize within-subject differences is preferred for the rmANCOVA.

**Table 2.** Main results with and without acuity as a covariate.

*RM-ANOVA: 0 and 90 degree relative angle between center and surround, and no surround conditions*			
Acuity as covariate?	Main effect of group	Main effect of condition	Group × condition interaction
Yes	F(4, 131) = 3.59, *p* = 0.01, η² = 0.099	F(1.91, 249.6) = 164.51, *p* < 0.001, η² = 0.557	F(7.62, 249.6) = 1.83, *p* = 0.08, η² = 0.053
No	F(4, 132) = 2.34, *p* = 0.06, η² = 0.066	F(1.91, 252.32) = 159.85, *p* < 0.001, η² = 0.548	F(7.65, 252.32) = 1.84, *p* = 0.07, η² = 0.053
*Model-fitted parameter ANOVAs*			
Acuity as covariate?	Group effect: M parameter	Group effect: o parameter	Group effect: w parameter
Yes	F(4, 123) = 2.19, *p* = 0.07, η² = 0.066	F(4, 123) = 4.95, *p* < 0.001, η² = 0.139	F(4, 123) = 1.06, *p* = 0.38, η² = 0.033
No	F(4, 124) = 3.23, *p* = 0.01, η² = 0.094	F(4, 124) = 5.11, *p* < 0.001, η² = 0.142	F(4, 124) = 1.01, *p* = 0.41, η² = 0.031

Bayes factors were computed (via anovaBF function in the BayesFactor R package; Morey & Rouder, 2021) to examine the strength of evidence for the null and alternative hypotheses to supplement classical null hypothesis tests. We computed these Bayes factors with the alternative model in the numerator and null model in the denominator and assessed the degree of group difference with acuity entered into the null model in the denominator. Although strict dichotomous decision boundaries are often anathema to Bayesian approaches, prior work tends to generally consider Bayes factors greater than 3 or less than ⅓ to be meaningful evidence in support of the null or alternative (depending on which hypothesis/model is in the numerator or denominator; Hoijtink, Mulder, van Lissa, & Gu, 2019).

To more fully characterize the dependence of suppression on relative orientation for each individual, we fit each participant's contrast decrement data for the 0 degrees, 20 degrees, 45 degrees, 70 degrees, and 90 degrees conditions to an exponential function: *P = −Me^−^^θ^^/w^ + o*. The three free parameters (M, w, and o) represent our dependent variables of interest: the offset parameter (o) represents orientation-insensitive (i.e. untuned) gain-control while the magnitude (M), and tuning width (w) parameters jointly characterize orientation-dependent suppression. Fitting was done in Python using scipy.optimize.curvefit nonlinear least squares fitting algorithm (Virtanen, Gommers, & Oliphant, 2020). Fits for individual participants were determined to be adequate if the variance of the data after subtracting the fit was lower than the variance of the raw data. By this criterion, only 9 of the 138 datasets were not well characterized by the exponential fit (1 PSZ, 3 BPD, 2 HC, 3 PSZrel, and 0 PBDrel). These nine subjects were excluded from all reported analyses in which the fit parameters were the dependent variable. Thus, it was concluded that the exponential function was an appropriate way of characterizing surround suppression behavior.

To generate hypotheses about the factors contributing to observed group differences in suppression of perceived target orientation as a function of surround orientation, a well-established divisive normalization model (Reynolds & Heeger, 2009) was adapted to simulate behavior on this dataset: *R = A_c_C_c_/(A_c_C_c_ + C_s_e^−^^θ^^/w^ +* σ*)*. The model equation and parameters used to generate the simulated suppression tuning curves are fully detailed in the discussion. In the original model, attention provides multiplicative enhancement of neuronal responses to stimuli: focal attention (*A_c_*) enhances only the target response; distributed attention would enhance both target and surround responses. If this multiplicative modulatory term (*A_c_*) is instead used to represent the more general concept of “amplification following segmentation,” then either low acuity or broadly distributed attention (or a combination of the two) will result in reduced amplification of the center (lower *A_c_* values) and therefore stronger divisive normalization (response suppression) by the surround. Further, widely reported deficits in cortical untuned gain control associated with schizophrenia (Butler, Silverstein, & Dakin, 2008; Uhlhaas & Singer, 2010; Yoon, Maddock, & Rokem, 2010) can be simulated by decreasing the semi-saturation constant in the denominator (σ). Quantitative fitting of the model to the data was not attempted; parameters were selected to illustrate how attention and suppression may interact to generate the patterns observed in the data.

## Results

The near and far surround conditions are expected to invoke different neuronal mechanisms of suppression; however, the far surround condition produced no significant modulation of perceived contrast in the present study and is therefore reported only in the Supplementary Material (Figure S1). In the near-surround condition, all groups showed strong suppression of perceived contrast in the presence of a parallel surround and weaker suppression by misaligned surrounds (see Figure 1).

A repeated measures analysis of covariance was run on the three key near surround conditions (0 degrees, 90 degrees, and no-surround as a baseline), with Huynh-Feldt correction (ε = 0.991) and visual acuity as a covariate. This revealed significant main effects of group and condition (F_group_(4, 131) = 3.59, *p* = 0.01, η² = 0.099; F_condition_(1.91, 249.6) = 164.51, *p* < 0.001, η² = 0.557; Supplementary Figure S2); however, the interaction of group and condition was not significant (F(7.62, 249.6) = 1.83, *p* = 0.08, η² = 0.053). A post hoc Bayes factor (BF) was computed to assess the lack of group differences observed for the 0 degrees condition while controlling for acuity by including acuity in the null hypothesis model. The 0 degrees BF was 0.001 (i.e. lack of group difference was 1000 times more likely). For comparison, the 90 degrees BF was 102.6 in favor of group differences.

**Figure 2.**
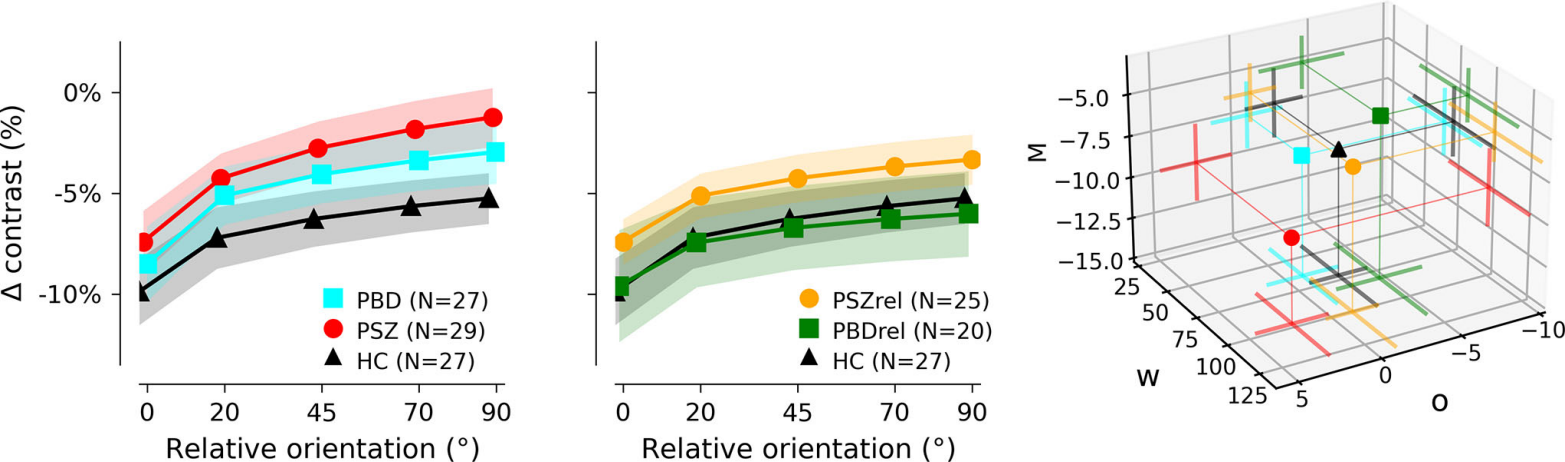
**Estimation of influence of orientation on surround suppression.** Each participant's data in the near condition was fit to an exponential function describing the dependence of surround suppression on relative orientation: *P = −Me^−^^θ^^/w^ + o*, where *M* represents modulation magnitude, *w* characterizes tuning width, and *o* estimates orientation-insensitive suppression that is present even at 90 degrees relative orientation. Datasets for which the fit did not decrease variance are excluded from the group averages shown in this figure (3 PSZrel, 3 PBD, 1 PSZ, 2 HC, and 0 PBDrel). *Points* indicate mean fit parameters; error bars/shading indicate 95% bootstrapped confidence intervals. *Far right*: Average values of fit parameters describing the sensitivity to orientation (*M*), the rate of decay of suppression as a function of relative orientation (*w*), and minimum suppression (*o*). PSZ showed greater sensitivity to orientation (more negative *M*), weaker untuned gain control (less negative *o*), and – along with PSZrel – a tendency toward broader orientation tuning of suppression (higher *w*).

Figure 2 characterizes the fitted exponential function (*P = −Me^−^^θ^^/w^ + o*) results for each group. Group differences in the three fit parameters were assessed with 1-way ANCOVAs, using acuity as a covariate. Previous work suggests that PSZ have broader orientation tuning (Rokem et al., 2011; Schallmo, Sponheim, & Olman, 2013), and PSZ and PSZrel did tend to have fits with larger *w* parameters (broader orientation tuning), but there was not a significant difference between groups (F(4, 123) = 1.06, *p* = 0.38, η² = 0.033). The *M* parameter showed a marginal effect of group that was not significant (F(4, 123) = 2.19, *p* = 0.07, η² = 0.066) when acuity was entered as a covariate, but was significant without acuity as a covariate (see Table 2). The untuned gain control *o* parameter exhibited the strongest group effect (F(4, 123) = 4.95, *p* < 0.001, η² = 0.139). Follow-up pairwise comparisons showed that PSZ exhibited less negative *o* parameters when compared both to HCs and to BPDrel (FDR corrected *p* values < 0.006).

**Figure 3.**
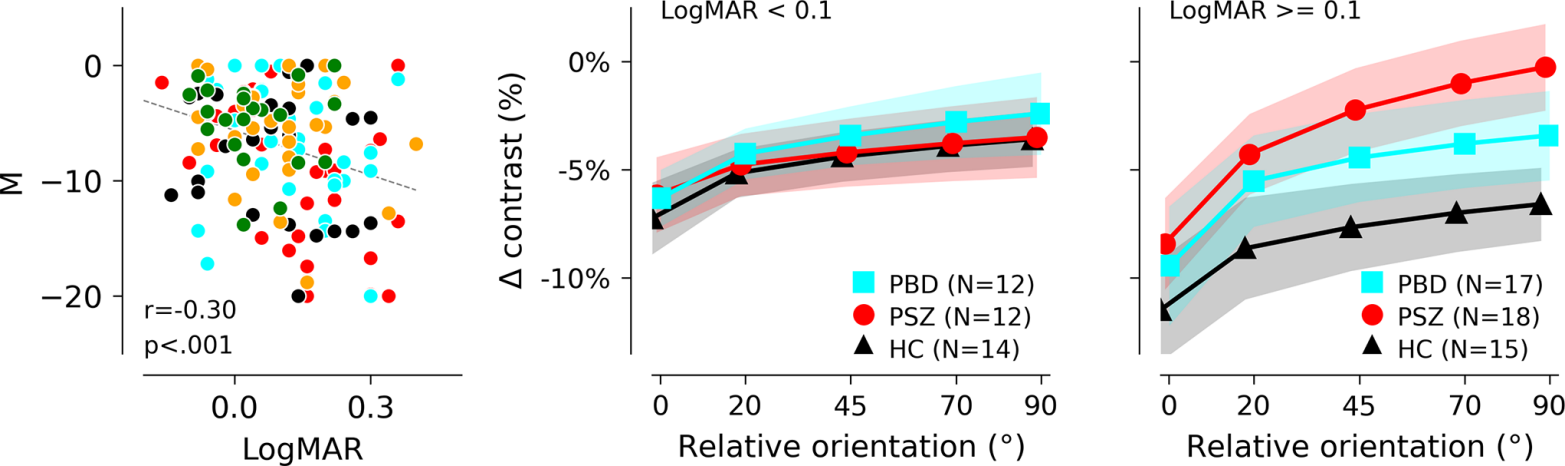
**Association between orientation-sensitivity of perceptual suppression and acuity.** *Left panel*: Across all participants, worse acuity (higher LogMAR scores) was associated with greater modulation (*M*) of suppression between parallel and orthogonal surround conditions. Right panels: to illustrate this effect, groups were split into sub-groups of participants with acuity at logMAR values of 0.1 or better (equivalent to Snellen acuity of 20/25) and participants with acuity measured less than LogMAR = 0.1. Averages of individual exponential fits to suppression as a function of surround orientation are plotted here, as in Figure 2, for controls and patient groups; for plots of average behavioral data, and data for relative groups, see Supplementary Figure S4.

Although there were no significant group differences in measured acuity (see Table 1), acuity moderated the relationship between diagnostic group and M in a stepwise manner (F(4, 119) = 2.96, *p* = 0.02, η² = 0.090; Baron & Kenny, 1986). To illustrate this moderation effect, Figure 3 depicts each group split by LogMAR acuity at 0.1 (Snellen acuity 20/25). This visualization shows that group differences are exaggerated in participants with low acuity. For HCs, reduced acuity was associated with stronger suppression in all stimulus conditions, whereas in PSZ, reduced acuity was associated with reduced suppression by orthogonal surrounds.

Finally, we conducted exploratory correlational analyses to test whether untuned gain control as measured by the *o* parameter tracked meaningfully with individual differences in atypical sensory experiences using the SGI. To avoid the psychometric pitfalls of sum scores (McNeish & Wolf, 2020), we conducted a four factor oblimin-rotated exploratory factor analysis (EFA) on the 36 item-level data and extracted Jos ten Berge factor score estimates using the psych R package (Revelle, 2021; Ten Berge, Krijnen, & Wansbeek, 1999). The four factor solution produced the most negative Bayesian Information Criterion (BIC; BIC = −1534.58) relative to the three and five factor solution suggesting the four factor solution best balanced model parsimony and fit which is consistent with previous work (Bailey, Moussa-Tooks, Klein, Sponheim, & Hetrick, 2021; Hetrick, Erickson, & Smith, 2012; χ²(492) = 886.05, *p* < 0.001, TLI = 0.86, CFI = 0.89, RMSEA = 0.08). Loadings greater than 0.3 for each factor are presented in Supplementary Table S1. Only the scores on the third factor correlated meaningfully with the offset parameter even before correction for multiple comparisons (r(122) = 0.19, uncorrected *p* = 0.037). This third factor loaded most heavily on over-inclusion items such as “I notice background noises more than other people” and “I seem to hear the smallest details of sound” although it also loaded onto a few perceptual-modulation items (e.g. “Sometimes I notice background noises more than usual”) and fatigue/stress items (e.g. “When I'm tired sounds seem amplified”). For BPRS, five factors were extracted based on the most negative BIC value (χ²(166) = 342.48, *p* < 0.001, TLI = 0.71, CFI = 0.83, RMSEA = 0.09). None of the factor score estimates for these factors correlated meaningfully with individual differences in the offset parameter.

## Discussion

The reduced suppression evident across near surround conditions (as demonstrated by the group differences in the offset parameter, *o*) for PSZ is consistent with previous reports of weakened untuned gain control associated with PSZ (Butler, Silverstein, & Dakin, 2008; Schallmo, Sponheim, & Olman, 2015). PBD and PSZrel exhibited intermediate deficits in untuned gain control relative to controls and PSZ. These intermediate deficits may be indicative of a spectrum of psychotic psychopathology in which PSZ are at one end, HCs are at the opposite end, and PBD and PSZrel sit in the middle. Further evidence for this perspective is the fact that PBD and PSZrel also exhibited intermediate levels of atypical sensory experiences (SGI) and general psychiatric symptoms (BPRS). Indeed, individual differences in over-inclusive perceptual experiences predicted weakened untuned gain control across participants; however, this was an exploratory association and the effect size of the relationship was small such that this observation needs to be replicated in an independent sample.

Evidence for atypical orientation-dependent suppression mechanisms in PSZ was mixed. The orientation-dependent parameters (i.e. *M* and *w)* were not significantly different between groups and we did not observe a significant interaction between group and condition. Having said this, post hoc Bayes factors provided strong evidence of a lack of difference between groups for the parallel-surround condition and strong evidence of group differences in the orthogonal condition. The preservation of suppression in the parallel-surround condition (i.e. lack of group difference) is surprising and unique to the specific configuration we used. Although we are unaware of this effect being observed previously in the context of surround suppression, analogous findings have been reported in the context of perceptual grouping tasks in which PSZ performed similar to controls when grouping cues were strongest (Silverstein, Burten, Essex, Kovács, Susmaras, & Little, 2009; Uhlhaas & Silverstein, 2005). Thus, it is possible that PSZ and PBD's normative suppression for parallel-surrounds reflects a floor effect in which all groups are able to adequately process visual context when contrast is matched and center and surround orientations are aligned. This effect may have been more salient due to the matching of contrasts between center and surround, as opposed to previously published studies in which center and surround contrasts differed (Barch, Carter, Dakin, Gold, Luck, Macdonald, Ragland, Silverstein, & Strauss, 2012; Dakin, Carlin, & Hemsley, 2005).

The dependence of contrast perception on relative orientation was moderated by acuity: surround suppression differences between patients and controls were all but eliminated when participants with lower acuity (Snellen acuity worse than 20/25) were excluded from analysis. It is noteworthy that approximately half of each of our experimental groups (patients and controls alike) had vision that was not corrected to normal (20/20) during the experiment. Our measurement of acuity was a simple Snellen eye chart at the 2-meter viewing distance that would be used for the task, with participants using any prescription lenses they had brought with them. It is possible that the relatively poor acuity across all groups was a consequence of the visual working distance being a poor match for the correction a given participant was using. To further complicate the matter, we did not observe significant differences in acuity between HC and PSZ as has been reported previously (however, see Pokorny et al., 2021). This may suggest that our convenience sample of HCs happened to have unusually poor acuity relative to the general population.

If there had been no effect of acuity in our dataset, the pattern of results shown in the full group averages (see Figures 1, 2) might have been explained by a difficulty of the PSZ group to deploy spatial attention. Although perceptual suppressive mechanisms are generally reduced for patients with schizophrenia, the same patients also experience a unique difficulty in allocating visual spatial attention or controlling attention (Barch, Carter, & Dakin, 2012). Focal spatial attention is known to reduce surround suppression (Flevaris & Murray, 2015; Poltoratski, Ling, McCormack, & Tong, 2017; Schallmo, Grant, Burton, & Olman, 2016; Zenger, Braun, & Koch, 2000). Thus, when scene segmentation cues are not strong (e.g. the parallel-surround condition, when surround and center have the same contrast), an elevation of suppression due to impairment of focal spatial attention for patients with schizophrenia could mask or counterbalance the generally observed surround suppression deficit. In other words, if impaired spatial attention had the greatest effect for stimuli with the weakest segmentation cues (in our experiment, the parallel surround), then deficits in untuned gain control for patients would emerge as relative orientation increased, and we would see the pattern shown in Figures 1 and 2: stronger modulation by orientation in patients than in controls. Previous experiments may not have detected this effect because the lower contrast of the central target relative to the surround provided a consistent, strong segmentation cue to help capture the spatial attention of all participants. Further exploration of this effect with a sample of patients with more severe symptomatology and cognitive impairment, and a rigorous assessment of spatial attention will be important for corroborating this spatial attention hypothesis.

Reduced acuity, on the other hand, could affect task performance by altering an individual's access to segmentation cues and thereby increasing the strength of suppression (because the V1-extrinsic mechanisms that rescue neuronal responses from suppression; Self, Peters, & Possel, 2016; would be absent when boundaries are not detected). A thin black ring, always present on the screen, not only delineated the region where a participant could expect to see the target in this experiment but also formed an explicit (although subtle) boundary between the target and the surround. With poor acuity, this ring would be less visible and might even (along with the small gray gap) blend into the target and surround, removing an explicit segmentation cue and resulting in stronger suppression (Lamme, Rodriguez-Rodriguez, & Spekreijse, 1999). This prediction matches the pattern observed for control participants: participants with low acuity showed stronger suppression of perceived contrast, compared to participants with high acuity, for all stimulus conditions. Thus, it is possible that removing this thin black ring would have led to more similar levels of suppression across levels of visual acuity in controls. However, doing so would remove the fiducial mark for the target location, increasing the spatial uncertainty and likely making it more difficult to allocate spatial attention.

**Figure 4.**
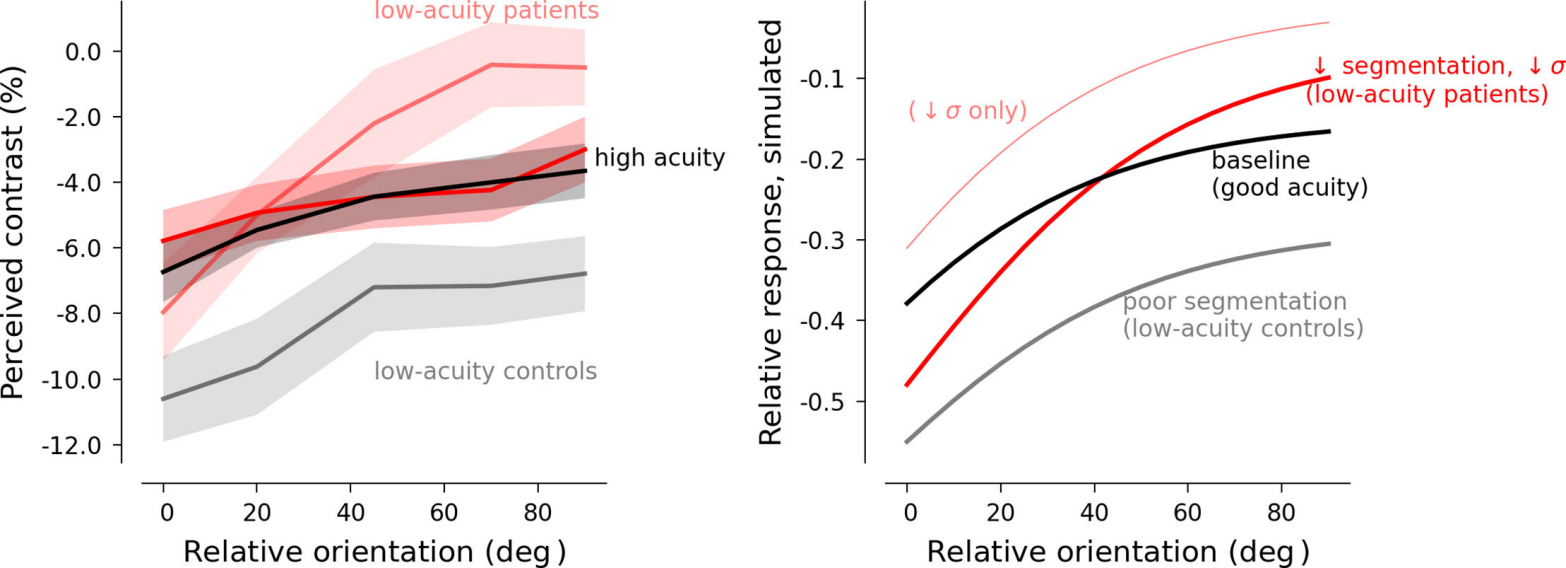
**Segmentation and untuned gain control can interact to determine orientation dependence of surround suppression.** Possible effects of attention and untuned gain control were simulated using a model styled after Reynolds and Heeger, 2009: *R = A_C_ C_C_***/**(*A_C_ C_C_* +*C_S_ e*^−^*^θ/w^* + σ) where *R* represents the normalized response to the central target (which predicts perceived contrast), *A_C_* represents amplification of the center relative to the surround, either by focal spatial attention or improved scene segmentation (possibly due to high acuity), *C_C_* represents the average neural drive from the central target, *C_S_* represents the drive from the surrounding stimulus, and σ is an additive constant that reflects nonspecific inhibition or untuned gain control. We modulate *C_S_* by an exponential term to reflect exponential dependence (*w*) of surround suppression on the relative orientation (*θ*) of the center and surround. For all simulations, *C_C_* and *C_S_* were held constant (0.8) to represent the equivalent contrast of the center and surround stimuli, and *w* was also fixed because the data did not provide strong evidence for group differences in the orientation tuning width of surround suppression. *Left panel*: Measured behavioral data (same data as in Supplementary Figure S4). *Right panel*: The *black line* simulates a baseline condition with good use of segmentation cues or focal attention and relatively strong cortical untuned gain control (*A_C_* = 2.0, σ = 0.4). The *gray line* simulates broadly distributed attention or low acuity (*A_C_* = 1.0), which results in a weakly orientation-dependent increase in the modeled strength of suppression compared to suppression during focal attention (*black line*). The *faint red line* simulates reduction of the semi-saturation constant (σ = 0.1), to simulate changes in untuned gain control associated with schizophrenia, which causes a reduction in suppression at all relative orientations (again with some orientation dependence because the relative magnitudes of *C_S_* and σ depends on the surround orientation). The *dark red line* shows that a combination of these two factors – orientation-dependent amplification of suppression by broadly distributed attention or poor acuity and reduction of baseline suppression by a reduced semi-saturation constant – produces the pattern observed in the behavioral data from PSZ with low acuity.

Stronger perceptual suppression due to poorer acuity (which we hypothesize reduces segmentation between center and surround) does not fully account for the observed relationship observed between acuity and suppression across all participants: whereas low-acuity HCs demonstrated greater suppression at all orientations, low-acuity PSZ showed reduced suppression for orthogonal surrounds resulting in greater sensitivity to orientation. Thus, to explain the full pattern of data, we would need to posit that (1) all participants experience increased suppression when acuity reduces segmentation cues, and (2) of the PSZ group, only individuals with low acuity experience reduced untuned gain control (orientation-insensitive inhibition). Figure 4 presents a computational model that simulates the measured pattern of responses in patients and controls.

Further work is necessary to determine the neural mechanisms underpinning this finding that acuity moderates the relationship between clinical group and suppression. The importance of acuity and retinal health in predicting schizophrenia has received increased attention recently, and rightly so (Hayes, Picot, Osborn, Lewis, Dalman, & Lundin, 2019; Silverstein, Fradkin, & Demmin, 2020; and references therein). Low acuity could have several causes, ranging from inadequate optical correction (a non-neuronal source) to retinal aberrations (i.e. altered function of the lateral inhibition that sharpens boundaries) to a reduction of the cortical suppressive mechanisms necessary for accurate delineation of object boundaries. We cannot, with the current dataset, distinguish among optical, retinal, or cortical sources for measured acuity in individuals nor address questions of whether acuity or retinal health is predictive of disease state in patients, although there are several known connections (Asanad, O'Neill, & Addis, 2021; Adams & Nasrallah, 2018; Moghimi, Torres Jimenez, & McLoon, 2020). Understanding the contributions of visual acuity to perceptual contrast surround suppression was not one of the original aims of the study, so the experimental protocol was not designed to ensure optimal correction or measure retinal health. Future studies with those controls in place will be necessary to pursue an understanding of the relationship between visual acuity and surround suppression in patients with psychosis.

Although other researchers have found group differences in acuity with evidence that the difference is neural in origin (Zemon, Herrera, Gordon, Revheim, Silipo, & Butler, 2021), we did not observe overall group differences in acuity in the current study. Our patient groups were outpatients with average IQ suggesting relatively normative levels of cognitive functioning. This too may have led to reductions in the magnitude of perceptual differences between controls and patients, given that links between higher IQ and stronger surround suppression have been reported among healthy adults (Arranz-Paraíso & Serrano-Pedraza, 2018; Cook, Hammett, & Larsson, 2016; Melnick, Harrison, Park, Bennetto, & Tadin, 2013; Troche, Thomas, Tadin, & Rammsayer, 2018). Further investigation in samples drawn from patients with greater cognitive impairment and greater functional deficits would be informative with respect to how illness severity impacts low-level visual deficits.

Within the framework of the normalization model discussed above, the intermediate performance of PSZrel could arise from a decrease in the semi-saturation term (σ) in the denominator used to regulate suppression (see red line in Figure 3A). In a recent study of contour integration, we found that contour detection performance of relatives was particularly robust against suppression by flanking context (Pokorny et al., 2021), to the point that their performance was superior to a control group. These findings together suggest that surround suppression might be subtly reduced by genetic liability for schizophrenia. However, a limitation of the current study is that the exclusion criteria for first degree relatives was less strict than for other groups due to the rare and valuable nature of the population. Thus, it is possible that differential recruitment for these groups introduced sampling bias.

Although the estimated orientation tuning of surround suppression was not significantly different between groups, a nonsignificant effect was observed that is consistent with previous studies reporting wider orientation tuning of suppressive mechanisms in PSZ (Rokem et al., 2011; Schallmo, Sponheim, & Olman, 2013). Broader orientation tuning associated with schizophrenia could arise either from weakened inhibitory mechanisms that refine orientation tuning of individual neurons in primary visual cortex (Ringach, Sapiro, & Shapley, 1997; Ringach, Shapley, & Hawken, 2002) or from broader tuning in higher-level grouping mechanisms (Shushruth et al., 2013). V1-intrinsic and V1-extrinsic mechanisms do not work independently: a less refined V1 representation of orientation could result in a greater likelihood of grouping visual features in extrastriate cortex (Schwartz, Sejnowski, & Dayan, 2009), which in turn would result in a higher likelihood of suppression. Difficulty allocating spatial attention could readily be either caused or confounded by broader tuning of suppressive mechanisms. Additional studies exploring the physiological basis of these behavioral effects will be necessary to tease apart the separate contributions of acuity, attention, and orientation tuning in early cortical visual networks.

The present work aimed to distinguish between possible neural mechanisms of impaired surround suppression in psychotic psychopathology. We found strong group differences in untuned (i.e. orientation-independent) suppression mechanisms while evidence for group differences in orientation-dependent suppression mechanisms was mixed. Unexpectedly, the association between group and orientation-dependent suppression was moderated by acuity. Given recent findings of surround suppression deficits in a variety of psychological disorders including schizophrenia, bipolar disorder, autism and depression (Salmela, Socada, & Söderholm, 2021; Schallmo, Sponheim, & Olman, 2015; Schallmo, Kolodny, & Kale, 2020), this work highlights the importance of understanding the causes of individual differences in visual acuity and how this relates to psychopathology.

## Supplementary Material

Supplement 1

Supplement 2

## References

[bib1] Adams, S. A., & Nasrallah, H. A. (2018). Multiple retinal anomalies in schizophrenia. *Schizophrenia Research,* 195, 3–12. 28755877 10.1016/j.schres.2017.07.018

[bib2] American Psychiatric Association, American Psychiatric Association Staff, American Psychiatric Association. (2000). Task Force on DSM-IV. *Diagnostic and Statistical Manual of Mental Disorders: DSM-IV-TR*. Washington, DC; American Psychiatric Association.

[bib3] Andreasen, N. C., Pressler, M., Nopoulos, P., Miller, D., & Ho, B. C. (2010). Antipsychotic dose equivalents and dose-years: a standardized method for comparing exposure to different drugs. *Biolical Psychiatry,* 67(3), 255–262. 10.1016/j.biopsych.2009.08.040 PMC3677042 19897178

[bib4] Arranz-Paraíso, S., & Serrano-Pedraza, I. (2018). Testing the link between visual suppression and intelligence. *PLoS One,* 13(7), e0200151. 29979774 10.1371/journal.pone.0200151 PMC6034845

[bib5] Asanad, S., O'Neill, H., Addis, H., et al. (2021). Neuroretinal Biomarkers for Schizophrenia Spectrum Disorders. *Translational Vision Science Technology,* 10(4), 29. 10.1167/tvst.10.4.29 PMC8083086 34004009

[bib6] Bailey, A. J., Moussa-Tooks, A. B., Klein, S. D., Sponheim, S. R., & Hetrick, W. P. (2021). The Sensory Gating Inventory-Brief. *Schizophrenia Bulletin Open,* 2(1), sgab019. 34414372 10.1093/schizbullopen/sgab019 PMC8369251

[bib7] Bair, W., Cavanaugh, J. R., & Movshon, J. A. (2003). Time course and time-distance relationships for surround suppression in macaque V1 neurons. *Journal of Neuroscience,* 23(20), 7690–7701. 12930809 10.1523/JNEUROSCI.23-20-07690.2003 PMC6740744

[bib8] Barch, D. M., Carter, C. S., Dakin, S. C., et al. (2012). The clinical translation of a measure of gain control: the contrast-contrast effect task. *Schizophrenia Bulletin,* 38(1), 135–143. 22101963 10.1093/schbul/sbr154 PMC3245599

[bib9] Baron, R. M., & Kenny, D. A. (1986). The moderator-mediator variable distinction in social psychological research: conceptual, strategic, and statistical considerations. *Journal of Personality and Social Psychology,* 51(6), 1173–1182. 3806354 10.1037//0022-3514.51.6.1173

[bib11] Bijanzadeh, M., Nurminen, L., Merlin, S., Clark, A. M., & Angelucci, A. (2018). Distinct Laminar Processing of Local and Global Context in Primate Primary Visual Cortex. *Neuron,* 100(1), 259–274.e4. 30220509 10.1016/j.neuron.2018.08.020 PMC6279245

[bib12] Boynton, G. M., Demb, J. B., Glover, G. H., & Heeger, D. J. (1999). Neuronal basis of contrast discrimination. *Vision Research,* 39(2), 257–269. 10326134 10.1016/s0042-6989(98)00113-8

[bib13] Butler, P. D., Silverstein, S. M., & Dakin, S. C. (2008). Visual perception and its impairment in schizophrenia. *Biological Psychiatry,* 64(1), 40–47. 18549875 10.1016/j.biopsych.2008.03.023 PMC2435292

[bib14] Carandini, M., & Heeger, D. J. (2011). Normalization as a canonical neural computation. *Nature Reviews Neuroscience,* 13(1), 51–62. 22108672 10.1038/nrn3136 PMC3273486

[bib15] Cavanaugh, J. R., Bair, W., & Movshon, J. A. (2002). Nature and interaction of signals from the receptive field center and surround in macaque V1 neurons. *Journal of Neurophysiology,* 88(5), 2530–2546. 12424292 10.1152/jn.00692.2001

[bib16] Chen, Y., Norton, D., & Ongur, D. (2008). Altered center-surround motion inhibition in schizophrenia. *Biological Psychiatry,* 64(1), 74–77. 18206855 10.1016/j.biopsych.2007.11.017 PMC2483430

[bib17] Cook, E., Hammett, S. T., & Larsson, J. (2016). GABA predicts visual intelligence. *Neuroscience Letters,* 632, 50–54. 27495012 10.1016/j.neulet.2016.07.053 PMC5054983

[bib18] Dakin, S., Carlin, P., & Hemsley, D. (2005). Weak suppression of visual context in chronic schizophrenia. *Current Biology: CB,* 15(20), R822–R824. 16243017 10.1016/j.cub.2005.10.015

[bib19] First, M. B., Spitzer, R. L., Gibbon, M., & Williams, J. B. W. (2002). *Structured Clinical Interview for DSM-IV-TR Axis I Disorders, Research Version,* Patient Edition. New York, NY: Structured Clinical Interview for DSM-I/P.

[bib20] Flevaris, A. V., & Murray, S. O. (2015). Attention Determines Contextual Enhancement versus Suppression in Human Primary Visual Cortex. *Journal of Neuroscience,* 35(35), 12273–12280. 26338337 10.1523/JNEUROSCI.1409-15.2015 PMC4556791

[bib21] Franken, T. P., & Reynolds, J. H. (2021). Border ownership selectivity in area V4 occurs first in infragranular layers. *Journal of Vision,* 21(9), 2259.

[bib22] Gold, J. M., Fuller, R. L., Robinson, B. M., Braun, E. L., & Luck, S. J. (2007). Impaired top-down control of visual search in schizophrenia. *Schizophrenia Research,* 94(1-3), 148–155. 17544632 10.1016/j.schres.2007.04.023 PMC1978542

[bib23] Gold, J. M., Robinson, B., Leonard, C. J., et al. (2018). Selective Attention, Working Memory, and Executive Function as Potential Independent Sources of Cognitive Dysfunction in Schizophrenia. *Schizophrenia Bulletin,* 44(6), 1227–1234. 29140504 10.1093/schbul/sbx155 PMC6192492

[bib24] Greenwood, T. A., Shutes-David, A., & Tsuang, D. W. (2019). Endophenotypes in Schizophrenia: Digging Deeper to Identify Genetic Mechanisms. *Journal of Psychiatric Brain Science,* 4(2), e20190005. 10.20900/jpbs.20190005 PMC6594566 31245629

[bib25] Hayes, J. F., Picot, S., Osborn, D. P. J., Lewis, G., Dalman, C., & Lundin, A. (2019). Visual Acuity in Late Adolescence and Future Psychosis Risk in a Cohort of 1 Million Men. *Schizophrenia Bulletin,* 45(3), 571–578. 29901774 10.1093/schbul/sby084 PMC6483575

[bib26] Hetrick, W. P., Erickson, M. A., & Smith, D. A. (2012). Phenomenological dimensions of sensory gating. *Schizophrenia Bulletin,* 38(1), 178–191. 20525773 10.1093/schbul/sbq054 PMC3245584

[bib27] Hoijtink, H., Mulder, J., van Lissa, C., & Gu, X. (2019). A tutorial on testing hypotheses using the Bayes factor. *Psychological Methods,* 24(5), 539–556. 30742472 10.1037/met0000201

[bib28] Kotov, R., Krueger, R. F., Watson, D., et al. (2017). The Hierarchical Taxonomy of Psychopathology (HiTOP): A dimensional alternative to traditional nosologies. *Journal of Abnormal Psychology,* 126(4), 454–477. 28333488 10.1037/abn0000258

[bib29] Lamme, V. A., Rodriguez-Rodriguez, V., & Spekreijse, H. (1999). Separate processing dynamics for texture elements, boundaries and surfaces in primary visual cortex of the macaque monkey. *Cerebral Cortex (New York, N.Y.: 1991),* 9(4), 406–413. 10426419 10.1093/cercor/9.4.406

[bib30] Linares, D., Amoretti, S., Marin-Campos, R., et al. (2020). Spatial Suppression and Sensitivity for Motion in Schizophrenia. *Schizophrenia Bulletin Open,* 1(1), sgaa045.

[bib31] Longenecker, J. M., Pokorny, V. J., Kang, S. S., Olman, C. A., & Sponheim, S. R. (2021). Self-reported perceptual aberrations in psychosis map to event-related potentials and semantic appraisals of objects. *Journal of Abnormal Psychology,* 130(7), 785–796. 34780232 10.1037/abn0000697

[bib32] Markon, K. E., Chmielewski, M., & Miller, C. J. (2011). The reliability and validity of discrete and continuous measures of psychopathology: a quantitative review. *Psychological Bulletin,* 137(5), 856–879. 21574681 10.1037/a0023678

[bib33] Mathalon, D. H., Heinks, T., & Ford, J. M. (2004). Selective attention in schizophrenia: sparing and loss of executive control. *The* *American Journal of Psychiatry,* 161(5), 872–881. 15121653 10.1176/appi.ajp.161.5.872

[bib34] McNeish, D., & Wolf, M. G. (2020). Thinking twice about sum scores. *Behavior Research Methods,* 52(6), 2287–2305. 32323277 10.3758/s13428-020-01398-0

[bib35] Melnick, M. D., Harrison, B. R., Park, S., Bennetto, L., & Tadin, D. (2013). A strong interactive link between sensory discriminations and intelligence. *Current Biology: CB,* 23(11), 1013–1017. 23707433 10.1016/j.cub.2013.04.053 PMC3702042

[bib36] Mély, D. A., Linsley, D., & Serre, T. (2018). Complementary surrounds explain diverse contextual phenomena across visual modalities. *Psychological Review,* 125(5), 769–784. 30234321 10.1037/rev0000109

[bib37] Moghimi, P., Torres Jimenez, N., McLoon, L. K., et al. (2020). Electoretinographic evidence of retinal ganglion cell-dependent function in schizophrenia. *Schizophrenia Research,* 219, 34–46. 31615740 10.1016/j.schres.2019.09.005 PMC7442157

[bib38] Morey, R. D., & Rouder, J. N. (2021). Bayes Factor: Computation of Bayes Factors for Common Designs. R package version 0.9.12-43, Available at: https://CRAN.R-project.org/package=BayesFactor.

[bib40] Overall, J. E., & Gorham, D. R. (1962). The Brief Psychiatric Rating Scale. *Psychological Reports,* 10(3), 799–812.

[bib42] Peirce, J. W. (2007). PsychoPy–Psychophysics software in Python. *Journal of Neuroscience Methods,* 162(1-2), 8–13. 17254636 10.1016/j.jneumeth.2006.11.017 PMC2018741

[bib43] Peirce, J. W. (2008). Generating stimuli for neuroscience using PsychoPy. *Frontiers in Neuroinformation,* 2, 10. 10.3389/neuro.11.010.2008 PMC2636899 19198666

[bib44] Pokorny, V. J., Espensen-Sturges, T. D., Burton, P. C., Sponheim, S. R., & Olman, C. A. (2020). Aberrant Cortical Connectivity During Ambiguous Object Recognition Is Associated With Schizophrenia. *Biological Psychiatry: Cognitive Neuroscience and Neuroimaging,* 6(12), 1193–1201. 33359154 10.1016/j.bpsc.2020.09.018 PMC8035333

[bib45] Pokorny, V. J., Lano, T. J., Schallmo, M. P., Olman, C. A., & Sponheim, S. R. (2021). Reduced influence of perceptual context in schizophrenia: behavioral and neurophysiological evidence. *Psychological Medicine,* 51(5), 786–794. 31858929 10.1017/S0033291719003751 PMC7444089

[bib46] Pokorny, V. J., & Sponheim, S. R. (2021). Neural Indicator of Altered Mismatch Detection Predicts Atypical Cognitive-Perceptual Experiences in Psychotic Psychopathology. *Schizophrenia Bulletin,* 48(2), 371–381. 10.1093/schbul/sbab127 PMC8886594 34665861

[bib47] Poltoratski, S., Ling, S., McCormack, D., & Tong, F. (2017). Characterizing the effects of feature salience and top-down attention in the early visual system. *Journal of Neurophysiology,* 118(1), 564–573. 28381491 10.1152/jn.00924.2016 PMC5511869

[bib48] Revelle, W. (2021). psych: Procedures for Psychological, Psychometric, and Personality Research. Northwestern University. Available at: https://CRAN.R-project.org/package=psych.

[bib49] Reynolds, J. H., & Heeger, D. J. (2009). The normalization model of attention. *Neuron,* 61(2), 168–185. 19186161 10.1016/j.neuron.2009.01.002 PMC2752446

[bib50] Ringach, D. L., Sapiro, G., & Shapley, R. (1997). A subspace reverse-correlation technique for the study of visual neurons. *Vision Research,* 37(17), 2455–2464. 9381680 10.1016/s0042-6989(96)00247-7

[bib51] Ringach, D. L., Shapley, R. M., & Hawken, M. J. (2002). Orientation Selectivity in Macaque V1: Diversity and Laminar Dependence. *Journal of Neuroscience,* 22(13), 5639–5651. 12097515 10.1523/JNEUROSCI.22-13-05639.2002 PMC6758222

[bib52] Rokem, A., Yoon, J. H., Ooms, R. E., Maddock, R. J., Minzenberg, M. J., & Silver, M. A. (2011). Broader visual orientation tuning in patients with schizophrenia. *Frontiers in Human Neuroscience,* 5, 127. 22069385 10.3389/fnhum.2011.00127 PMC3208208

[bib53] Salmela, V., Socada, L., Söderholm, J., et al. (2021). Reduced visual contrast suppression during major depressive episodes. *Journal of Psychiatry Neuroscience,* 46(2), E222–E231. 33703869 10.1503/jpn.200091 PMC8061742

[bib54] Schallmo, M. P., Grant, A. N., Burton, P. C., & Olman, C. A. (2016). The effects of orientation and attention during surround suppression of small image features: A 7 Tesla fMRI study. *Journal of Vision,* 16(10), 19. 10.1167/16.10.19 PMC5015919 27565016

[bib55] Schallmo, M. P., Kolodny, T., Kale, A. M., et al. (2020). Weaker neural suppression in autism. *Nature Communications,* 11(1), 2675. 10.1038/s41467-020-16495-z PMC7260360 32472088

[bib56] Schallmo, M. P., Sponheim, S. R., & Olman, C. A. (2013). Abnormal contextual modulation of visual contour detection in patients with schizophrenia. *PLoS One,* 8(6), e68090. 23922637 10.1371/journal.pone.0068090 PMC3688981

[bib57] Schallmo, M. P., Sponheim, S. R., & Olman, C. A. (2015). Reduced contextual effects on visual contrast perception in schizophrenia and bipolar affective disorder. *Psychological Medicine,* 45(16), 3527–3537. 26315020 10.1017/S0033291715001439 PMC4624017

[bib58] Schütt, H. H., Harmeling, S., Macke, J. H., & Wichmann, F. A. (2016). Painfree and accurate Bayesian estimation of psychometric functions for (potentially) overdispersed data. *Vision Research,* 122, 105–123. 27013261 10.1016/j.visres.2016.02.002

[bib59] Schwartz, O., Sejnowski, T. J., & Dayan, P. (2009). Perceptual organization in the tilt illusion. *Journal of Vision,* 9(4), 19.1–20. 10.1167/9.4.19 PMC2852324 19757928

[bib60] Self, M. W., Peters, J. C., Possel, J. K., et al. (2016). The Effects of Context and Attention on Spiking Activity in Human Early Visual Cortex. *PLoS Computational Biology,* 14(3), e1002420. 10.1371/journal.pbio.1002420 PMC4807817 27015604

[bib61] Serrano-Pedraza, I., Romero-Ferreiro, V., Read, J. C. A., et al. (2014). Reduced visual surround suppression in schizophrenia shown by measuring contrast detection thresholds. *Frontiers in Psychology,* 5, 1431. 25540631 10.3389/fpsyg.2014.01431 PMC4261701

[bib62] Seymour, K., Stein, T., Sanders, L. L. O., Guggenmos, M., Theophil, I., & Sterzer, P. (2013). Altered contextual modulation of primary visual cortex responses in schizophrenia. *Neuropsychopharmacology,* 38(13), 2607–2612. 23842600 10.1038/npp.2013.168 PMC3828531

[bib63] Shushruth, S., Nurminen, L., Bijanzadeh, M., Ichida, J. M., Vanni, S., & Angelucci, A. (2013). Different orientation tuning of near- and far-surround suppression in macaque primary visual cortex mirrors their tuning in human perception. *The Journal of Neuroscience : the Official Journal of the Society for Neuroscience,* 33(1), 106–119. 23283326 10.1523/JNEUROSCI.2518-12.2013 PMC3711542

[bib64] Sillito, A. M., & Jones, H. E. (2002). Corticothalamic interactions in the transfer of visual information. *Philosophical Transactions of the Royal Society of London. Series B, Biological Sciences,* 357(1428), 1739–1752. 12626008 10.1098/rstb.2002.1170 PMC1693075

[bib65] Silverstein, S. M., Berten, S., Essex, B., Kovács, I., Susmaras, T., & Little, D. M. (2009). An fMRI examination of visual integration in schizophrenia. *Journal of Integrative Neuroscience,* 8(2), 175–202. 19618486 10.1142/s0219635209002113

[bib66] Silverstein, S. M., Fradkin, S. I., & Demmin, D. L. (2020). Schizophrenia and the retina: Towards a 2020 perspective. *Schizophrenia Research,* 219, 84–94. 31708400 10.1016/j.schres.2019.09.016 PMC7202990

[bib67] Tadin, D., Kim, J., Doop, M. L., et al. (2006). Weakened center-surround interactions in visual motion processing in schizophrenia. *Journal of Neuroscience,* 26(44), 11403–11412. 17079669 10.1523/JNEUROSCI.2592-06.2006 PMC6674537

[bib68] Ten Berge, J. M. F., Krijnen, W. P., & Wansbeek, T. (1999). Some new results on correlation-preserving factor scores prediction methods. *Linear Algebra and its Appllications,* 289(1-3), 311–318.

[bib69] Tibber, M. S., Anderson, E. J., & Bobin, T., et al. (2013). Visual surround suppression in schizophrenia. *Frontiers in Psychology,* 4, 88. 23450069 10.3389/fpsyg.2013.00088 PMC3584288

[bib70] Troche, S. J., Thomas, P., Tadin, D., & Rammsayer, T. H. (2018). On the relationship between spatial suppression, speed of information processing, and psychometric intelligence. *Intelligence,* 67, 11–18.

[bib71] Uhlhaas, P. J., & Silverstein, S. M. (2005). Perceptual organization in schizophrenia spectrum disorders: empirical research and theoretical implications. *Psychological Bulletin,* 131(4), 618–632. 16060805 10.1037/0033-2909.131.4.618

[bib72] Uhlhaas, P. J., & Singer, W. (2010). Abnormal neural oscillations and synchrony in schizophrenia. *Nature Reviews: Neuroscience,* 11(2), 100–113. 20087360 10.1038/nrn2774

[bib73] Virtanen, P., Gommers, R., Oliphant, T. E., et al. (2020). SciPy 1.0: fundamental algorithms for scientific computing in Python. *Nature Methods,* 17(3), 261–272. 32015543 10.1038/s41592-019-0686-2 PMC7056644

[bib74] Wechsler, D. (1997). *Manual for the Wechsler adult intelligence scale—third edition (WAIS-III)*. San Antonio, TX: The Psychological Corporation.

[bib75] Wichmann, F. A., & Hill, N. J. (2001). The psychometric function: I. Fitting, sampling, and goodness of fit. *Perception Psychophysics,* 63(8), 1293–1313. 11800458 10.3758/bf03194544

[bib76] Xing, J., & Heeger, D. J. (2001). Measurement and modeling of center-surround suppression and enhancement. *Vision Research,* 41(5), 571–583. 11226503 10.1016/s0042-6989(00)00270-4

[bib77] Yang, E., Tadin, D., Glasser, D. M., Hong, S. W., Blake, R., & Park, S. (2013). Visual context processing in schizophrenia. *Clinical Psychological Science,* 1(1), 5–15. 23997995 10.1177/2167702612464618 PMC3756604

[bib78] Yang, E., Tadin, D., Glasser, D. M., Wook Hong, S., Blake, R., & Park, S. (2013). Visual context processing in bipolar disorder: a comparison with schizophrenia. *Frontiers in Psychology,* 4, 569. 24009596 10.3389/fpsyg.2013.00569 PMC3757289

[bib79] Yoon, J. H., Maddock, R. J., Rokem, A., et al. (2010). GABA concentration is reduced in visual cortex in schizophrenia and correlates with orientation-specific surround suppression. *Journal of Neuroscience,* 30(10), 3777–3781. 20220012 10.1523/JNEUROSCI.6158-09.2010 PMC2846788

[bib80] Zemon, V., Herrera, S., Gordon, J., Revheim, N., Silipo, G., & Butler, P. D. (2021). Contrast sensitivity deficits in schizophrenia: A psychophysical investigation. *The European Journal of Neuroscience,* 53(4), 1155–1170. 33118212 10.1111/ejn.15026

[bib81] Zenger, B., Braun, J., & Koch, C. (2000). Attentional effects on contrast detection in the presence of surround masks. *Vision Research,* 40(27), 3717–3724. 11090664 10.1016/s0042-6989(00)00218-2

[bib82] Zenger-Landolt, B., & Koch, C. (2001). Flanker effects in peripheral contrast discrimination—psychophysics and modeling. *Vision Research,* 41(27), 3663–3675. 11712981 10.1016/s0042-6989(01)00175-4

[bib83] Zhaoping, L. (2005). Border ownership from intracortical interactions in visual area v2. *Neuron,* 47(1), 143–153. 15996554 10.1016/j.neuron.2005.04.005

