# Supplementary material for: Weakened untuned gain control is associated with schizophrenia while atypical orientation-tuned suppression depends on visual acuity
Source: J Vis. 2023 Feb 1;23(2):2. doi: 10.1167/jov.23.2.2 (PMC9904333; doi:10.1167/jov.23.2.2)
Supplement: Supplement 2 [file jovi-23-2-2_s002.pdf]

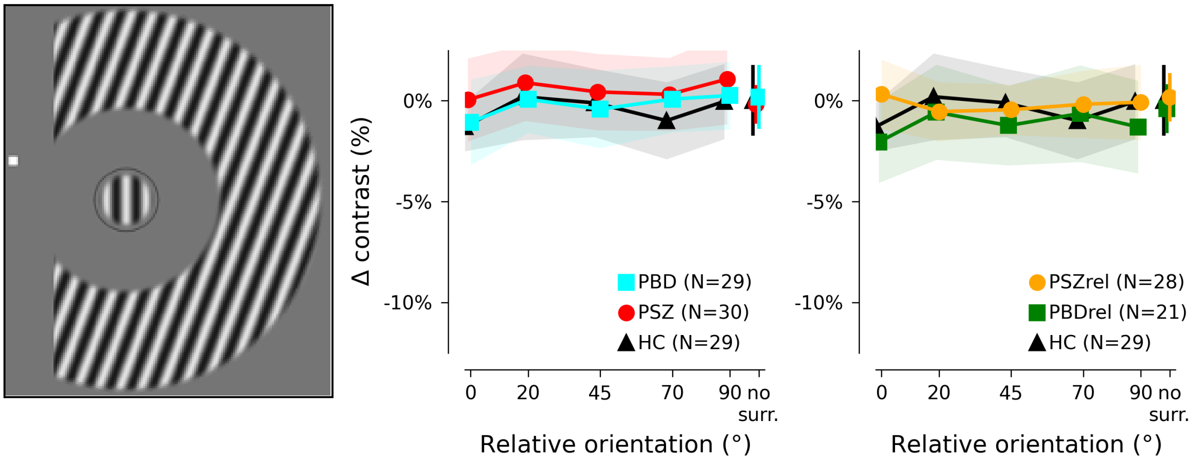

Figure S1. Results of far surround condition. To investigate previous reports that near and far surround stimuli produced suppression via different neuronal mechanisms, a far surround condition was included in the task design. Trial structure and analysis were identical to methods reported for the near surround condition. Not even in the control population do our results replicate the findings of Shushruth et al., 2013, in which the magnitude of perceived contrast suppression by the far surround was comparable to the magnitude of suppression by the near surround, and suppression was orientation-sensitive in both conditions. The most significant difference between this study and the previous study is that stimuli in the previous study were centered at 6 degrees eccentricity instead of 3 (grating spatial frequency and stimuli radii were similarly scaled by roughly 2X). Suppression mechanisms vary systematically across the visual field, typically growing stronger in the periphery, which is the most likely explanation for the difference between our results and previous results.

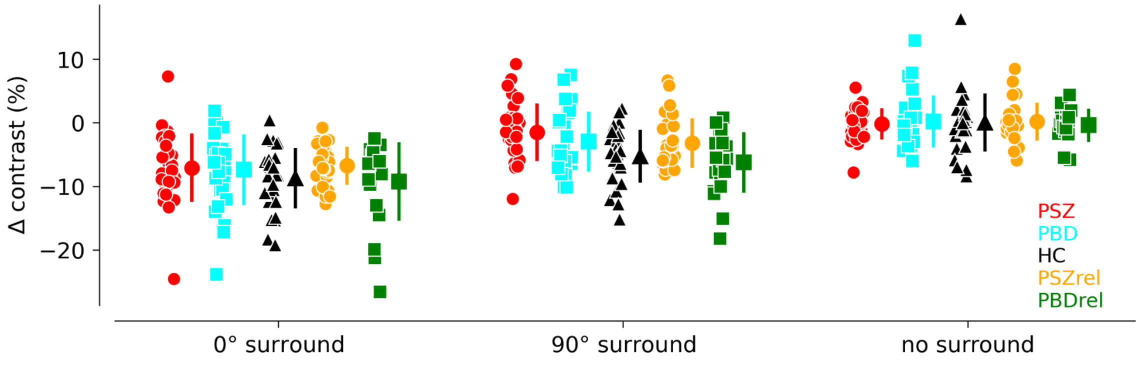

Figure S2. Data used for initial statistical tests of group differences in suppression. Effects were first assessed by testing performance on three conditions for each group: parallel surround, orthogonal surround, and no surround. These data points are plotted here so the reader can visually confirm the effects; smaller points are behavioral values from individual participants; large dots are group means; error bars indicate standard deviation.

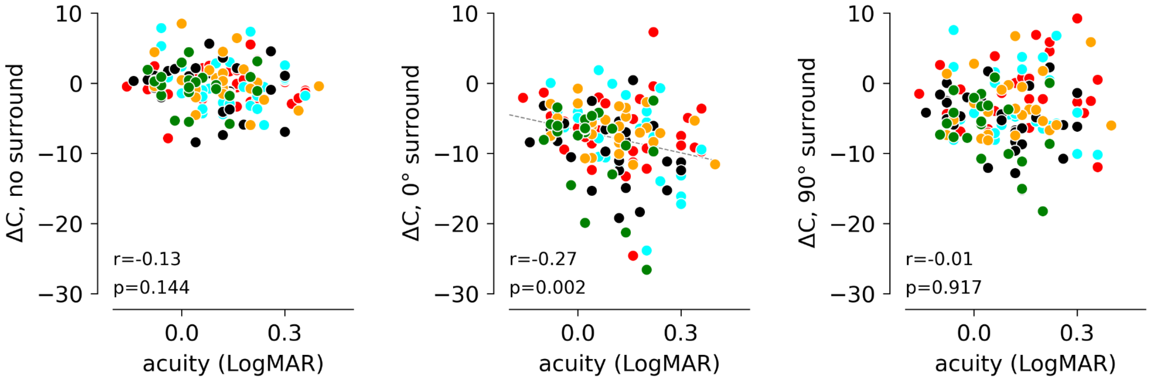

Figure S3. Across all groups, participants with worse acuity show stronger surround suppression in the parallel-surround condition. Each point represents an individual subject; mapping of color to group membership is the same as in other figures. Similar plots shown in Fig. 3 of the main manuscript show the relationship between acuity and M, the magnitude of modulation between the 0° and 90° surround conditions. Uncorrected p-values are shown.

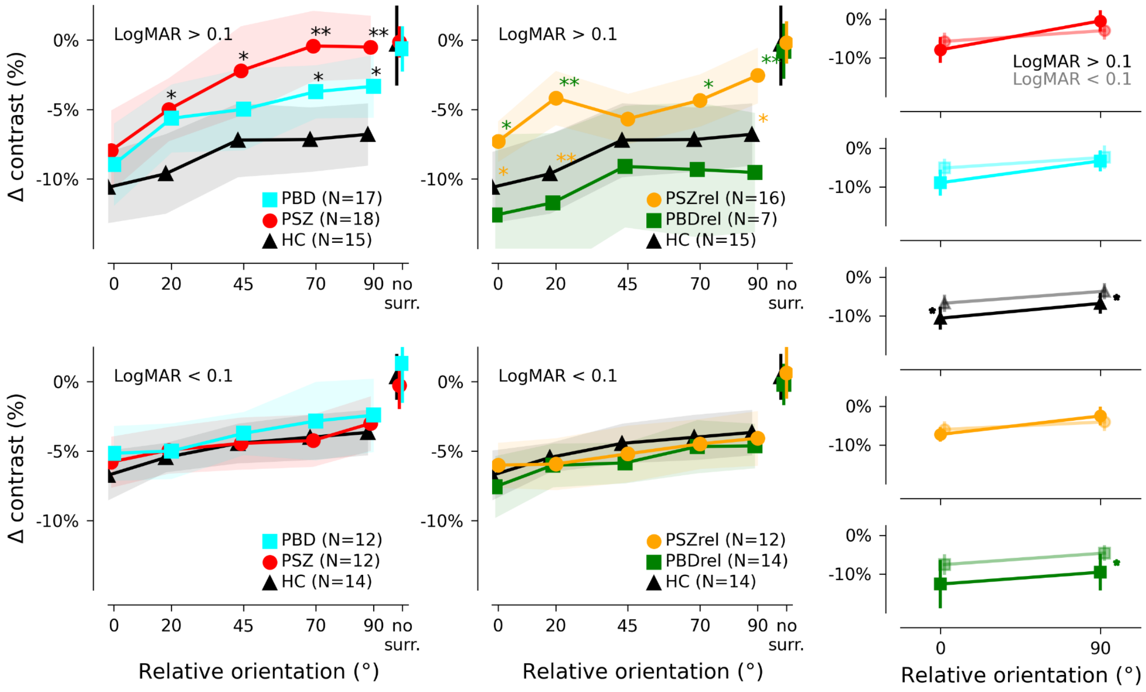

Figure S4. Split-half illustration of association between acuity and perceptual suppression. Figure 3 in the main manuscript shows the averaged exponential fits to individual datasets, and only for patients and controls. Here, raw behavioral data are averaged for patients, controls, and relative groups. Overall trends are the same: no differences in high-acuity sub-populations and strong differences in low-acuity groups, although variability in the smaller relative groups precludes drawing any conclusions. At far right, the extreme conditions are illustrated, illustrating the uniform shift of suppression with acuity in the HC group, and the interaction between suppression and acuity for PSZ. * indicates $p < 0.05$ for post-hoc paired t-tests, uncorrected. These analyses are intended for hypothesis development only.

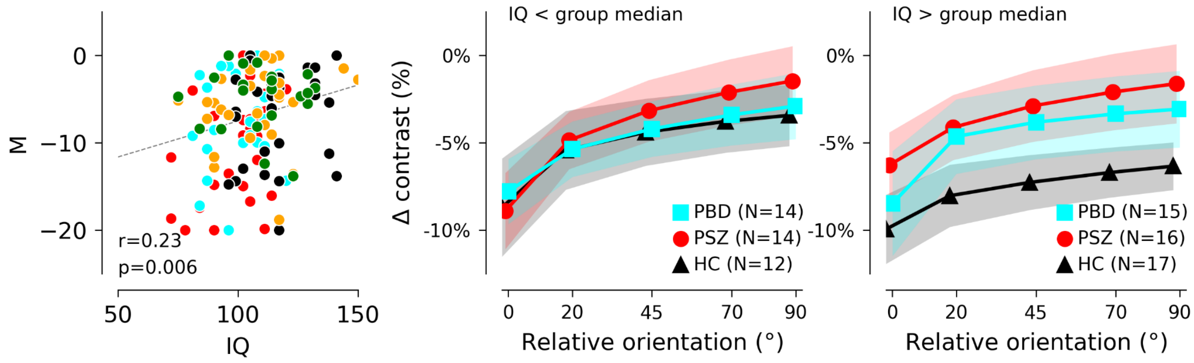

Figure S5. Comparisons between task performance and IQ. Because our preliminary analyses of behavioral data indicated a group difference in IQ (Table 1), and because earlier studies have reported an association between IQ and perceptual surround suppression [72–75,81], we also investigated the association between IQ and task performance. While, across all groups, IQ did show a relationship with *M* (left panel: higher IQ predicts less dependence of suppression on orientation, after regressing out potential effects of acuity), IQ did not significantly moderate the relationship between group and *M* ($F(4,119)=0.24$, $p=0.91$, $\eta^2=.008$). Right panels: An exploratory split-halves analysis indicates that patients with higher IQ tended to have weaker surround suppression. Groups were split at the median of each group to illustrate the association between *M* and Estimated IQ. Median values for the groups shown here were 105, 105, and 114 for PSZ, PBD, and CTRL. Brief discussion: High performance on catch trials, and successful parametric manipulation of perceptual performance within individuals, indicates that this association is not simply due to a generalized cognitive deficit. While more work will be required to provide a clear answer to the question of how Estimated IQ is associated with performance on surround suppression tasks, we have considered several possible reasons for the association. Estimated IQ was measured by WAIS Vocabulary and visual Block Design (shape) tasks. The spatial imagery skills required to perform well on the block design task are housed in parietal cortex, which is also implicated as a key region for allocating spatial attention. Thus, focal spatial attention could be related to WAIS performance. An alternative mechanism by which IQ and suppression tasks might be related involves alterations in inhibition throughout the brain. In V1, altered excitation/inhibition balance is thought to produce atypical contrast surround suppression; in other brain regions, deficient inhibition may result in altered sensory and cognitive processing (e.g., reduced sensory gating, impaired mismatch detection, selective attention deficits and difficulty maintaining working memory [23,24,82,83]). Through this mechanism, performance on surround suppression tasks would be correlated with, but not directly related to, the neural mechanisms resulting in reduced IQ.

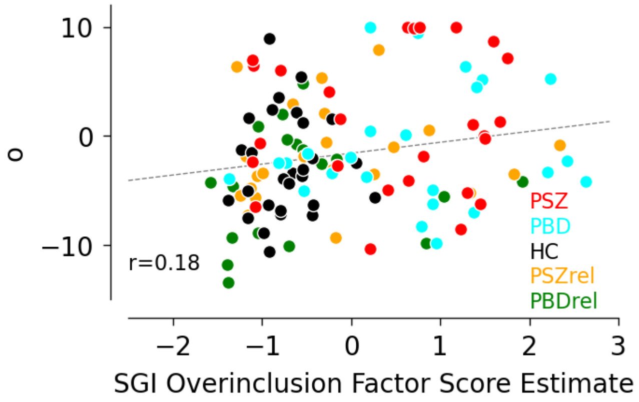

Figure S6. Exploratory correlation between SGI Overinclusion factor score estimates and offset parameters. Factor score estimates were derived using the ten Berge method which preserves the correlations between factors in the factor score estimates. The factor score indeterminacy (i.e. the correlation between the factor score estimates and the true factor scores) for the SGI overinclusion factor was .952.
